# Multidisciplinarity in Transition Pathways for Patients With Kidney Disease: The Current State of Play

**DOI:** 10.3389/fped.2021.689758

**Published:** 2021-09-20

**Authors:** Dorella Scarponi, Viviana Cammaroto, Andrea Pasini, Claudio La Scola, Francesca Mencarelli, Cristina Bertulli, Marco Busutti, Gaetano La Manna, Andrea Pession

**Affiliations:** ^1^Nephrology and Dialysis Unit, Department of Pediatrics, IRCCS Azienda Ospedaliero-Universitaria of Bologna, Bologna, Italy; ^2^Nephrology, Dialysis and Transplantation Unit, Department of Experimental, Diagnostic and Specialty Medicine, IRCCS Azienda Ospedaliero-Universitaria of Bologna, Bologna, Italy

**Keywords:** transition, kidney, chronic, multidisciplinary, pediatric

## Abstract

In the field of medical care, successful transition from pediatric-centered to adult-oriented healthcare can provide a sense of continuity in the development of youth, and prepare them to accept responsibility for and manage their own chronic kidney condition in complete autonomy. The so-called transition process requires the presence of some basic aspects: a multidisciplinary team, which acts as a bridge between child and adult services; a comprehensive clinical, cognitive, psychological, and social change for the young people; the involvement of family and caregivers. Within the framework of transition and chronicity during the developmental age, we selected international papers explaining models which agreed on some important steps in the transition process, although many differences can be observed between different countries. In fact, in Europe, the situation appears to be heterogeneous as regards certain aspects: the written transition plan, the educational programmes, the timing of transfer to adult services, the presence of a transition coordinator, a dedicated off-site transition clinic. We then analyzed some studies focusing on patients with renal diseases, including the first to contain a standardized protocol for transition which was launched recently in the USA, and which seems to have already achieved important positive, although limited, results. In Italy, the issue of transition is still in its infancy, however important efforts in the management of chronic kidney disease have already been initiated in some regions, including Emila Romagna, which gives us hope for the future of many young people.

## Introduction

### An Overview of Transitional Care

The term *transitional care* is a heterogeneous concept which incorporates a series of steps aimed at ensuring the coordination and continuity of care for patients who are transferred from one centre to another or between different levels of intensity of care (1–3). The starting point is the integration of the various professional roles, settings, and healthcare pathways involved. Therefore, the model of “intermediate care” provides a bridge, for example between the hospital and the home, or between pediatric and adult services, within which two very different settings synergistically create a process of total patient care (1, 4, 5). Transition, therefore, implicates the passage from a child-centered to an adult-centered health system, with the aim of preparing adolescents to take responsibility for and manage their own health problems (6–8). Due to the progressive increase in the number of adolescents suffering from chronic diseases and the higher survival rates of a large number of children and adolescents affected by chronic diseases necessitating special care (SiQuAs-VRQ, 2014), the concept of *transitional care* has become ever more emergent over the last few years. In Italy, it is estimated that around 15–18% of adolescents suffer from a chronic illness; of these, ~8-900,000 have only one disease, while circa 100-150,000 have at least two (9). Among these, according to the National Plan for Chronicity (PNC) elaborated by the Italian Ministry of Health (10), per year, there are ~30–50 new cases requiring dialysis, with the prospect of rapid access to transplantation. Since the concept of *transitional care* was creating terminological confusion in the scientific literature, the Italian Society for Quality Healthcare (SiQuAs-VRQ) organized a project-event called “Transitional Care” in 2014, which was aimed at defining a precise topic of interest, namely the transition of healthcare for patients with chronic diseases from the pediatric to the adult age.

In order to have a better understanding of the current state of transitional care, we searched the PubMed database for articles relevant to the topic: the transition of adolescents and young adults with chronic diseases to adult services; we then created a subgroup of articles related specifically to adolescents and young adults with chronic or end-stage renal disease, with or without a transplant, who were “in transition” to adult services. As numerous articles related to chronic diseases were available, we narrowed our selection criteria by including only the articles which considered the following: studies which had applied the existing guidelines in the planning and implementation of their transition process (more specifically, if there was a written/verbal protocol to follow, a specialized transition team, dedicated transition clinics, involvement of specific roles i.e., a psychologist/psychotherapist, a coordinator etc.); studies representative of the main countries where the international transition guidelines for pediatric patients have been applied; studies which evaluated the efficacy of transition from a medical (adherence, transplant success, reduced hospitalization), psycho-social (readiness, degree of satisfaction, quality of life) and logistic (dedicated structures, costs, feasibility) point of view; studies which considered participation in “camps” as a measure of effectiveness of the transition process; previous reviews of retrospective studies which included the abovementioned criteria. We excluded the articles which did not contain pertinent information: studies which evaluated the outcomes of pediatric patients after their transition to adult services, not during the process; studies which were similar in terms of authors, results and country of origin; studies which had not applied the international guidelines for the transition of pediatric patients; studies with only one outcome (graft loss or hospitalization or medical adherence) without considering psycho-social factors (readiness, degree of satisfaction, quality of life), costs or feasibility; retrospective studies which provided scanty information (data collection, sample size, outcomes, etc.). We selected 37 articles based on the abovementioned criteria and excluded nine.

### Chronicity During the Developmental Age

The management of chronic illnesses differs greatly between adolescent and adult patients. Primarily, the constantly changing needs of patients as they develop creates healthcare complexities requiring medical care and social welfare interventions, possibly long-term, which are organized on the basis of personalized plans and a combination of primary and hospital care. Furthermore, it is possible that the disease and/or disability can cause oftentimes irreversible developmental delay, which needs to be prevented. Finally, the need to facilitate the inclusion of youth with chronic diseases in academic, recreational, and social situations constitutes an important component of welfare interventions (10). For this reason, the PNC supports the empowerment of all of the parties involved, in such a way as to help patients and their families through the acquisition of competences and trust, so that the patient as a “person” becomes expert in navigating their personal history of “coexistence” with chronicity (10). The presence of a multi-professional team comprising doctors, nurses, dieticians, social workers, play therapists, psychologists, and educators has been seen to be the most efficacious way of minimizing their disability and maximizing their potential. The support that children and their families receive from a multi-professional team, rather than from a single treating physician, is the main difference between pediatric and adult healthcare (11).

### Transition: A Challenge for the Adolescent

For youth with chronic diseases, the transition pathway toward adulthood represents an additional complexity. The difficulty in treating adolescent patients with chronic diseases lies in the series of their physical, psychological, and emotional changes, which complicate the management of the transition as a process of care. In fact, for many children and their families, this developmental transition, which begins at around 13 to 14 years of age and continues until late adolescence or early adulthood, can be an especially stressful process, particularly because the illness makes the adolescents more dependent on their parents and healthcare staff, which hinders their physiological individuation. Consequently, one of the first reactions we see in clinical practice is oppositional behavior accompanied by outbursts of anger and the resulting lack of treatment adherence from patients (12, 13).

### Guidelines and Assessment Tools

#### What the Literature Tells Us About the Transition of Patients With Chronic Diseases

In its most desirable form, the transfer of a pediatric patient from a model of pediatric care initially managed by the caregiver to the self-management of the disease in an adult context is an event which occurs at the end of a process (7). We compared the different guidelines available for the management of the care pathway during the transition of chronic patients in general (Table 1), namely those from the American Academy of Pediatrics (AAP) (15, 16) and the United Kingdom National Institute for Health and Care Excellence (NICE) (14), which include recommendations concerning both the preceding and successive phases of the transfer itself. In particular, the AAP (16) has developed an approach known as “Six Core Elements of Health Care Transition,” which can be applied to different care models. The six elements include:

1. Transition policy;
2. Transition tracking and monitoring;
3. Transition readiness;
4. Transition planning;
5. Transfer and/or integration into adult-centered care;
6. Transition completion and ongoing care with adult clinician.

**Table 1:** Guidelines for the transition from pediatric to adult healthcare.

	**ISN/IPNA (8)**	**NICE (14)**	**AAP (15, 16)**	**PNC (10)**
Period in which to initiate transition	Between 12 and 14 years	Between 13 and 14 years	Between 12 and 14 years	No specific age is indicated
Transition plan	Transition plan individualized according to the needs, abilities and competences shown at a specific age. Education involves the family and significant others Assessment of the readiness for transition Medical passport	Focus on the positive resources and possible objectives to be reached Person-centered approaches	Assess readiness for transition by means of a person-centered approach Address age-appropriate transition issues Work under a model of adult healthcare from the age of 18years	Basic training in compliance motivation and collaboration integrated multidisciplinary approach Favor independence in relation to the degree of maturity of the subject Increase awareness at school, during physical-sports and recreational activities
Timing of transfer to adult healthcare services	During a stable phase in the life of the adolescent/young adult After having completed compulsory education	During a period of relative stability for the young person The exact moment of transfer should be discussed with the young person	The transfer itself should happen between the ages of 18 and 21, even though it is possible before	No specific time for transfer is indicated
Personnel involved	Process managed by physicians (transition champions) and transition coordinators from the both the pediatric and adult services	A single professional allocated according to the young person's needs	Physicians from the pediatric and adult teams and a coordinator	The specialized pediatric unit and the pediatrician should integrate with the equivalent adult service and facilitate the handover of the young person
Specific physician	Yes - Nephrologist	No	Yes – Pediatrician specialized in youth with special healthcare needs	Yes – Specialist pediatrician in charge of coordinating the activities
External transition clinic	No, clinics should be included in adult services	No	No	No

Furthermore, the PNC highlights the “lack of clear indications regarding the process of ‘transition’ from the pediatric to the adult age or the age at which said transition should occur. On the other hand, there are no shared indications concerning the fact that all pediatric patients should be followed exclusively by pediatric centers and not by adult services.” Nevertheless, the lines of intervention for the management of pediatric chronicity must consider the peculiar characteristics related to childhood. Thus, the PNC envisages the identification of four macro activities:

(1) continuity of care for children with chronic health conditions;
(2) the role of the family;
(3) age-specific relational environments (school, sport, social);
(4) the passage from pediatric to adult management (10).

The different guidelines contain some common characteristics pertaining to the nature and phases of the transition process, which can be considered useful in the approach to nephrological patients too: start planning the transition process (14–17), transfer to the adult service (17). All the recommendations focus on the importance of the presence of a transition coordinator: the AAP guidelines, NICE, and PNC (10) refer to social workers, but make no specifications for the inclusion of psychologists and/or psychotherapists in the transition team. Furthermore, it is estimated that only around half of the European centres have a formalized transition care unit (18). Although programmes envisage high resource intensity, the development and running costs of a multidisciplinary transition programme can be compensated for by the positive outcomes of the patients involved (19).Transition should begin as soon as possible, with gradual processes of support, education, and preparation (14).

Multidisciplinary teams within dedicated clinics are able to help youth deal with the situations connected to transition (20). However, only a few of the guidelines make reference to this (8).

The lack of communication between hospital healthcare teams, patients, and primary care providers (usually pediatricians) is still a cause for concern (21). As regards transplanted children, who generally have a lifelong care plan to follow, dedicated transition appears inevitable. The medical passport represents an attempt to solve such problems by permitting the identification of individuals at risk, preventing premature transition, and improving long-term results (20).

#### What the Literature Tells Us About the Transition of Patients With Renal Disease

In nephropathic patients, some particular forms of treatment, such as dialysis, transplantation, and the risk of acute rejection and graft loss (22, 23) require long periods of hospitalization, isolation from peers, and interrupted schooling, which prolong the form of attachment to reference figures characterized by dependence on adults. In some cases, advanced renal disease can be associated with reduced cognitive capacity and increased psychological distress (24, 25). Of note is the increased risk of graft failure associated with older age (26); in fact, renal transplantation in adolescents and young adults has a poorer outcome compared to younger age groups (27). Transition plays an important role during this developmental period (28, 29). A child with kidney disease should therefore be treated by a multidisciplinary clinical and surgical team with specific pediatric competences, able to create a treatment plan comprehensive of conservative and nutritional requirements and corrective actions for associated anomalies and, at a later stage, dialysis and transplantation in an adequate setting (10).

Until well into 2016, the published articles on *transitional care* were subdivided according to their field of application: diabetes, arthritis, cystic fibrosis. Only over the last decade, have research efforts focused on analyzing the phenomenon of transition in the pediatric populations affected by kidney diseases, as described in the selected papers summarized in Table 2. In The UK, Harden et al. (36) performed a study aimed at evaluating the efficacy of the transition process involving youth and young adults with kidney disease; in Canada, Prestidge et al. (19) described a positive transition experience in terms of costs, transplantation, and survival of patients with a kidney transplant. Tong and collaborators (31) tried to develop a new transition clinic (YAC), based on the Oxford Kidney Unit model (36). The qualitative results suggest that the learning of self-management strategies through peer groups, encouragement, and a sense of responsibility can strongly motivate the patient toward autonomy. The possibility to conduct group activities in locations outside the clinical setting can also reduce the young person's health anxiety. Other studies included the degree of patient satisfaction in their indices of outcome (30, 33), concluding that in order to achieve a good transition result, planning must begin as soon as possible and monitoring must continue after transition is complete (30, 37). In particular, Chaturvedi et al. (30) suggest involving young patients in the planning phase of their transition. In Germany, the same result was obtained with pediatric renal transplant populations (34, 38). The abovementioned study was part of the TRANSNephro trial, the first randomized controlled trial in Germany and Austria to focus on transitional care. The objectives of the transition process for children were centered on self-care, autonomy, and treatment adherence (38). Although the majority of the centres involved offered educational programmes, none of them utilized tools for assessing their transition readiness (35). In the TRANSNephro study, age at transition was 18 years, yet most centres requested a special dispensation to extend pediatric care beyond that age; the reasons were medical in nature (transplant) and/or psychosocial (family, school, professional training). The aspects considered as fundamental for transition suitability included: autonomy, a sense of responsibility, emotional stability, cognitive maturity, knowledge of the disease and adherence to treatment. The role of emotional stability was defined as a prerequisite for acting responsibly and maturely, the lack of which was considered a risk factor. The key question should not be “How old is the patient?” but “How ready is the patient to transfer to adult healthcare?” (39).

**Table 2:** Characteristics of healthcare transition for adults and youth with chronic kidney disease.

**Country/study**	**Program/clinical population**	**Time of transfer**	**Transition coordinator**	**Transition clinic**	**Social worker/ psychologist/** **psychotherapist**	**Results**
Australia, Chaturvedi et al. (30)	– Youth with a kidney transplant (*N* = 11)	18–19 years, average age 19.5 (patient age range 18–23 yrs	The transition process is coordinated by a transition nurse (a nephrology appointment).	A dedicated transition clinic within the hospital with a pediatric and adult nephrologist.	No	Most of the patients remained clinically stable during the 2-year study period, as shown by the serum creatinine values pre- and post-transfer; no episodes of graft rejection or hospitalization.
Australia, Tong et al. (31)	– Youth with chronic kidney disease (N = 15)	Not specified (patient age range 18–26 yrs)	Not specified.	An external renal clinic for young adults (YAC) developed on the basis of the Oxford Kidney Unit reported by (32) and located in a community-based young adult healthcare center.	Social worker	No significant improvement in quality of life or adhesion to medication among the young adults following two multidisciplinary transition clinic appointments.
Canada, Prestidge et al. (19)	– Youth with a kidney transplant: study group (*N* = 12) vs. control group (*N* = 33)	From 16 years of age, though the time of transfer depends on the fact that the youth are clinically stable, willing, and deemed to be ready by the multidisciplinary team.	A clinic coordinator plans the first appointments in the adult unit and follows the participants' attendance.	Multidisciplinary transition clinic supported by a transition team.	Social worker and young adult health specialist	The transfer was a positive experience in terms of costs, transplantation, and patient survival; 2 years after transfer, there was a reduction in mortality (no episodes of rejection or graft loss) in the transition group, while costs were the same between the two groups.
Germany, Austria, Pape et al. (33)	– Youth with a kidney transplant (*N* = 66)	18 years	Three types of doctor: (1) A specialist adult nephrologist, (2) Surgeons or nephrologists in a private clinic (3) Different adult nephrologists in a private clinic.	Three types of clinic:(1) Transition clinic for young adults,(2) General adult transplantation clinic(3) Nephrologist.	No, data collected retrospectively.	No significant difference in graft survival among the three transition models; the patients in setting 1 were more satisfied and their medication was modified less; 50% of patients were satisfied with the pre- transfer education program.
Germany, Austria, Prüfe et al. (34)	TRANSNephro Youth with a kidney transplant (*N* = 111)	18 years in 16/21 centers, but most centers requested an extension of pediatric healthcare beyond 18 years (average age 18.3 years; range 16.5–36.7 years).	No, patients and their families were asked to find an adult nephrologist when the time for transfer was approaching.	No, patients and their families were asked to find a transplant center when the time for transfer was approaching, if necessary.	No. Contrary to the pediatric units, adult nephrology clinics did not provide the services of a psychologist or psychotherapist and the waiting list for psychotherapy in the community was up to 6–9 months.	The transition situation is notably heterogeneous and differs significantly among centers: The concept of transition is introduced quite late; most centers do not use a transition plan drawn up to contextualize their actions but rely instead on instinct and experience; there is a rigid age criterion which does not account for the needs and readiness of the patients; in some units, it is possible to find dedicated staff (champions) who coordinate transition, but this is not a widespread phenomenon; 86% of centers offer general internal training courses and recommend participating in educational programs; no combined clinics for adolescents and young adults are present, nor is there an integrated multidisciplinary approach, combined with peer support, to optimize specialized care.
The Netherlands, Sattoe et al. (32)	Camp COOL Youth with chronic kidney disease (*N* = 32)	16 years or older (up to 25 years).	No. “Buddies”, young patients who have already transitioned to adult healthcare, run the daily program, manage the camp and advise the participants who have yet to make the transition to adult care: their role is to help break the ice and initiate conversation, share their experiences and take care of the attendees: a proactive combination of supervisor, consultant and leader	No	No	The camp, through peer support, seems to have positive effects on the ability of youth with chronic kidney disease to self-manage their condition before transfer: more self-confidence, greater awareness of their condition, the desire to be more responsible and open to others and to have the courage to “stand on their own two feet”.
The United Kingdom Ghazanfar et al. (35)	– Youth with a kidney transplant: study group (*N* = 78) vs. control group (*N* = 58)	Extract from congress proceedings: no further information available about the full text.	Extract from congress proceedings: no further information available about the full text.	Transition clinic	Extract from congress proceedings: no further information available about the full text.	Significant improvement in patient and graft survival following the initiation of the transition process.
The United Kingdom Harden et al. (36)	Oxford-London Youth with a kidney transplant: study group (*N* = 12) vs. control group (*N* = 9)	Study group 18 years (16-18 years) transferred to a new integrated service Vs. control group 17.5 years (16–18 years) transferred directly to an adult nephrology service.	A youth worker acts as a bridge between the healthcare team and the patients, coordinating the clinic day, which involves an “ice-breaking” session: their role involves facilitating introducing new young adult patients to the organization of group events in order to encourage peer interaction and rebuild self-esteem.	An external integrated multidisciplinary transition service: a dedicated clinic for young adults was moved outside the hospital to a student college and a sports center in the center of Oxford in order to provide a normal environment for the young adults catalyse peer interaction between the patients.	No	Reduction in the rate of graft loss and acute rejection in the study group; no significant change in the standard immunosuppressive protocols
Switzerland, Weitz et al. (37)	– Youth with a kidney transplant: study group with a transition program (*N* = 26) vs. control group without a transition program (*N* = 33)	The transition program begins at 14 years of age; at 16 years, the time of transition is defined based on psychosocial development and self-management skills.	Not specified	Structured transition clinic supported by a multidisciplinary team of healthcare professionals.	Social worker	A standardized multilevel transition program started early seems to improve the clinical status of the transplanted patients: episodes of acute rejection reduced by a third and a slower decline in estimated glomerular filtration rate in the study group, compared to controls, 3 years after transition.
USA, Raina et al. (20)	Literature review and survey among pediatric nephrologists in the USA. Youth with a kidney transplant from 49 centers	16–18 years on half of the centers, 16 years or younger in the other half.	The staff involved in the transition process varies widely according to the practices of different centers: most are pediatric nephrologists (40/94), adult nephrologists (34/49) and social workers (27/49).	Only 23% of centers made use of a dedicated clinic.	Very few of the people interviewed had seen a psychologist.	There is a notable heterogeneity in clinical practice in The USA: variability of the staff involved in the transition process, a lack of dedicated clinics, and incoherent use of patient-centered tools (transition questionnaires and evaluation of readiness) and inefficient communication between the pediatric and adult teams, as well as with primary care providers.

The International Society of Nephrology together with the International Pediatric Nephrology Association (ISN/IPNA) elaborated specific guidelines for the transition of pediatric patients with kidney disease, approved for implementation in the United Kingdom, The USA, Australia, and Egypt. In Italy, the PNC elaborated by the Ministry of Health (10) compiled an initial list of chronic diseases, for the majority of which, at present, there are no specific national plans of action.

Recently, a group of American researchers (20) conducted a literature review and a survey among pediatric nephrologists in The USA, with the objective of analyzing the current state of transitional care (Table 2). This led to the creation of the transition protocol “RISE to transition,” for patients with a kidney transplant. The protocol is aimed at improving: graft survival, compliance to treatment, and quality of life. It comprises four areas of competency that the patient must reach before the moment of transition:

1. Recognition - awareness of their disease process, the healthcare system and the reason for transplant;
2. Insight - awareness of their own emotional needs;
3. Self-reliance - autonomous treatment planning and participation;
4. Establishment - healthy lifestyle choices, life-long adherence to medications and follow-up, the acquisition of psychosocial skills.

Competency in these areas forms the basis for effective transition. The key players are: the patient and family; the pediatric team; the transition team; the adult team; the primary care provider. The protocol also refers to:

1. The medical passport.
2. Complete medical records.
3. Medical records relating to the transplant.
4. Transition readiness assessment, performed every 6 months.

The transition process is divided into three stages, depending on patient age:

1. Pre-transition stage (14–18 years): dedicated to the education of the family.
2. Active transition stage (18–21 years): the pediatric team initiates the transition process, assessing the needs and competences of the patient and family in order to personalize the process. The transition team assesses patient progress in terms of the four areas of competency and acts as a bridge between the pediatric and adult services. During this period, assessments are carried out every 6 months until the patient is deemed autonomous.
3. Post-transition stage (21–26 years): the adult team becomes the primary team. Six months after transition, a meeting between the two teams is planned to discuss the patient's progress, and this is repeated until the patient is 26 years of age (20). As this is a complex phenomenon, the current national transitional care project (SIQuAS-VRQ, 2014) was created with the objective of delivering homogeneous care during the passage from the pediatric to the adult age and was developed according to the best practice guidelines proposed by Donabedian (40): active listening, the ability to work in a team, organizational quality, and relational quality. The typology of care must also take into consideration the long history of the disease and the considerable experience each patient has in dealing with healthcare services and professionals (SIQuAS-VRQ, 2014). The project, which is consistent with the PNC guidelines, is articulated in three phases which proceed in parallel:

1. The establishment of a “Control Group” and a Technical Advisory Committee, comprising various medical scientific societies and health charities, whose duty it is to construct hypotheses regarding the functional and quality requirements of a “transition service”;
2. The mapping of the experiences present on the territory by means of a questionnaire aimed at gathering information regarding the current management of transitional care;
3. The collection of the experimentations of the regional realities (Piedmont, Marche and Apulia) which have been instrumental in defining concrete organizational models.

Currently, there are four nosological areas of interest (oncology, diabetology, rare diseases and respiratory diseases), which are the subject of experimentation in the three aforementioned Italian regions. To these, cystic fibrosis, nephrology, and rheumatology have been added.

The most frequently studied aspect concerns the “taking charge” of the patients, using multiprofessional and interdisciplinary modalities, with the aim of building a real “transition group” which plans the “transition service.”

In this phase, the investigative activities involve:

(1) *The centrality of the subjects*;
(2) *The concept of transition* viewed as a long-term process;
(3) *The relevance of the temporal dimension* of the illness experience;
(4) *The co-construction of therapeutic relationships and diagnostic and care pathways*.

If transition is to be successful, it is important to evaluate the readiness of the young person and their family. Different tools exist for use in the pediatric populations affected by kidney disease:TRxANSITION Scale (41); Youth Quiz from the On Trac programme (42); Transition Readiness Assessment Questionnaire (TRAQ) (43); Readiness for Transition Questionnaire (RTQ) (43). In agreement with the general principles of the ISN/IPNA guidelines (2011), the TRxANSITION Scale (41) has been considered suitable for the evaluation and monitoring of patient and caregivers' progress in terms of reaching the objectives of transition through 10 domains (Type of illness, Medications, Adherence, Nutrition, Self-management, Informed-reproduction, Trade/school issues, Insurance issues, Ongoing support, New health providers). Based on the patient and caregivers' responses on the TRxANSITION Scale, the multidisciplinary team decides the most suitable time for transition.

## Summary

Transitional care is an essential step for patients with kidney disease. In Europe, there are numerous heterogeneous general models for approaching transitional care for patients with chronic diseases, so we decided to evaluate them in terms of their healthcare-related and organizational aspects, and how these aspects could be better integrated in the field of nephrology. The most evident differences include the timing of transfer, the presence of a coordinator, and the logistics. The greatest limitation, which reduces their applicability, is the absence of written guidelines. The American experience can be seen as one of the first good examples of transition for patients suffering from kidney disease and although there are inevitable differences born from specific context-related cultural characteristics, it must be considered a milestone. In Italy, the situation appears to be considerably heterogeneous and, in many respects, disorganized. However, the transition process has already been activated in at least three regions, including Emila-Romagna, and has been applied to various chronic diseases of the developmental age, with the same objectives: the inclusion and empowerment of the patient, and the same multidisciplinary structure.

It is evident that, to date, transitional care is not only an objective to reach, but a process still in its developmental stages that has to deal with the restrictions and limits imposed by the different legislative procedures and organization of healthcare services seen in different countries, as well as within individual territories. Nonetheless, we agree on the four areas of competency that the patient must reach before the moment of transition: awareness of their disease process, awareness of their own emotional needs, autonomous treatment planning and participation, healthy lifestyle choices.

We know with certainty that when the guidelines are applied it is possible to increase the effectiveness criteria: quality of life, survival, compliance and reduce drop-out. Furthermore, the degree of patient satisfaction in their indices of outcome rises.

Within this reference framework, the healthcare objectives of patients with chronic diseases, which cannot include recovery, concern the overall definition of the clinical picture, functional state, control of symptoms, prevention of disability and improvement in quality of life. In order to achieve this, it is necessary to redefine the care pathways able to take charge of the patient in the long term, guaranteeing continuity of care and the integration of social and health interventions by both the pediatric and adult services.

## Author Contributions

DS and VC contributed to the conception and design of the work, perfomed the literature search, and wrote the first draft of the manuscript. APa, GL, and APe contributed to the conception and design of the work, and critically reviewed the manuscript. CB, CL, FM, and MB critically reviewed the manuscript. All authors contributed to the article and approved the submitted version.

## Conflict of Interest

The authors declare that the research was conducted in the absence of any commercial or financial relationships that could be construed as a potential conflict of interest.

## Publisher's Note

All claims expressed in this article are solely those of the authors and do not necessarily represent those of their affiliated organizations, or those of the publisher, the editors and the reviewers. Any product that may be evaluated in this article, or claim that may be made by its manufacturer, is not guaranteed or endorsed by the publisher.

## Abbreviations

SiQuAs-VRQ: Società Italiana per la Qualità dell'Assistenza Sanitaria
UNICEF: United Nations International Children's Emergency Fund
PNC: Piano Nazionale della Cronicità
AAP: American Academy of Pediatrics
NICE: United Kingdom National Institute for Health and Care Excellence
ISN: International Society of Nephrology
IPNA: International Pediatric Nephrology Association
USA: United States of America
ONU: Organizzazione delle Nazioni Unite
TRAQ: Transition Readiness Assessment Questionnaire
RTQ: Readiness for Transition Questionnaire
YAC: Young Adult Clinic
COOL: Communicatie (Communication), Ontplooiing (Self-development), Ontmoeting (Meeting), Lol (Fun).
